# MNRR1, a Biorganellar Regulator of Mitochondria

**DOI:** 10.1155/2017/6739236

**Published:** 2017-06-08

**Authors:** Lawrence I. Grossman, Neeraja Purandare, Rooshan Arshad, Stephanie Gladyck, Mallika Somayajulu, Maik Hüttemann, Siddhesh Aras

**Affiliations:** Center for Molecular Medicine and Genetics, Wayne State University School of Medicine, 540 E. Canfield Ave., Detroit, MI 48201, USA

## Abstract

The central role of energy metabolism in cellular activities is becoming widely recognized. However, there are many gaps in our knowledge of the mechanisms by which mitochondria evaluate their status and call upon the nucleus to make adjustments. Recently, a protein family consisting of twin CX_9_C proteins has been shown to play a role in human pathophysiology. We focus here on two family members, the isoforms CHCHD2 (renamed MNRR1) and CHCHD10. The better studied isoform, MNRR1, has the unusual property of functioning in both the mitochondria and the nucleus and of having a different function in each. In the mitochondria, it functions by binding to cytochrome *c* oxidase (COX), which stimulates respiration. Its binding to COX is promoted by tyrosine-99 phosphorylation, carried out by ABL2 kinase (ARG). In the nucleus, MNRR1 binds to a novel promoter element in *COX4I2* and itself, increasing transcription at 4% oxygen. We discuss mutations in both MNRR1 and CHCHD10 found in a number of chronic, mostly neurodegenerative, diseases. Finally, we propose a model of a graded response to hypoxic and oxidative stresses, mediated under different oxygen tensions by CHCHD10, MNRR1, and HIF1, which operate at intermediate and very low oxygen concentrations, respectively.

## 1. Introduction

The coiled-coil-helix-coiled-coil-helix domain- (CHCHD-) containing proteins are small, nuclear-encoded proteins that are characterized by four cysteine residues organized in twin cysteine motifs, where the cysteines are separated by nine amino acids (twin CX_9_C proteins). They were initially thought to localize only to the intermembrane space (IMS) of the mitochondria, into which at least some have been shown to be imported via the Mia40/Erv1 relay system, although some of them have since been found in the nucleus [1–3]. This review will focus on two members of the twin CX_9_C protein family, the isoforms CHCHD2/MNRR1 and CHCHD10, that are turning out to have surprisingly far ranging effects on mitochondrial function.

The twin CX_9_C family is characterized by the CHCH domain [4], which contains a helix-turn-helix fold, where each helix contains the CX_9_C motif [5–7]. The structure of the CHCH domain was resolved by protein-folding studies for Cox17, which is another CHCH protein [5, 6]. These cysteine-containing motifs help to stabilize twin alpha helices by forming disulfide bonds between the cysteine residues [8]. Although each protein in the family contains the above elements, they are unique in other aspects including their size, other structural elements, and their functions (see [9] for review).

The discovery of the Mia40 (CHCHD4)/Erv1 import pathway in the mitochondrial intermembrane space (IMS) heightened interest in the CHCHD-containing protein family [10–13]. Unlike matrix or inner-membrane-bound proteins, proteins that use the Mia40 pathway do not require a mitochondrial-targeting sequence (MTS) precursor. Import via Mia40 works through a disulfide relay system wherein Mia40 is anchored to the inner mitochondrial membrane, facing into the IMS. In this system, CX_9_C proteins are brought into the IMS from the cytosol via the translocase of the outer membrane (TOM) in a reduced, unfolded state. The oxidized cysteine residues of Mia40 then form disulfide bridges with the cysteine residues of the incoming twin CX_9_C protein. After further modification to the disulfide bridges, the imported CX_9_C protein is released into the IMS and Mia40 is reoxidized by Erv1 [13]. It is interesting to note that some CHCHD-containing proteins are predicted to have an MTS; these include CHCHD1, CHCHD2, and CHCHD10 [9]. Such observations suggest that these proteins can use the translocase of the inner-membrane (TIM)/TOM import as an alternative route or that they may be able to also localize to the mitochondrial matrix (or the inner mitochondrial membrane). A third possibility is that these presequences are not functional since MNRR1/CHCHD2 has been shown to localize to the mitochondria even after the removal of its MTS (Aras and Grossman, unpublished data).

CX_9_C proteins were initially well characterized in *Saccharomyces cerevisiae*. The systematic analysis of the full complement of the CX_9_C protein family by Longen and others [8] revealed that 13 of the 14 putative family members identified were highly conserved from yeast to mammals. A genome-wide analysis of CX_9_C proteins in eukaryotes [14] expanded information on this protein family. Twin CX_9_C proteins were found to be conserved across the organisms included in the study, except for three obligate intercellular parasites that contain mitosomes. The evolutionary conservation across organisms containing true mitochondria suggested that these CX_9_C proteins are important in mitochondrial function and, indeed, members of this family operate as subunits in complexes I and IV of the electron transport chain (ETC), as cytochrome *c* oxidase (COX) assembly factors, and they participate in mitochondrial protein import, structure, and function. Some of the proteins also cluster into groups of unknown functions [14]. In this review, we will focus on two of these proteins that have very recently been associated with neurodegenerative diseases, MNRR1/CHCHD2 and CHCHD10. CHCHD2 was recently renamed as Mitochondrial Nuclear Retrograde Regulator 1, MNRR1 [3], which will be used from here on.

**Figure 1:**
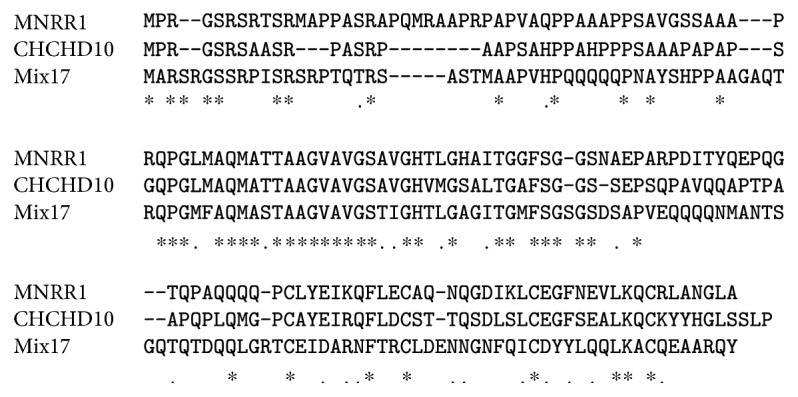
Alignment of human CHCHD10, human MNRR1, and yeast Mix17. Identical residues (^∗^) and similar residues (.) are indicated.

MNRR1 and CHCHD10 have a common ancestor in yeast, Mix17p (formerly known as Mic17p). Both mammalian proteins are 42% conserved with Mix17p. On a screen of deletion mutants for all twin CX_9_C proteins in yeast to identify their role, the deletion of Mix17 decreased oxygen consumption to ~50% of WT [8]. Mix17 was originally characterized by Huh et al. to be located in the nucleus [15]. Gabriel et al. [16] considered the possibility that the presence of the GFP tag used in the Huh study interfered with the localization of the protein and hence characterized the localization of endogenous Mix17. Besides the nucleus, they found that Mix17 is localized to the mitochondrial IMS and is imported via the Mia40 pathway [16]. Mix17 appeared to be a stress sensitive protein whose levels increase in response to treatment with chemicals that induce DNA replication stress [17]. The same study also characterized changes in protein localization in response to the stress. However, this study used GFP-tagged proteins and hence raises the possibility that stress-induced Mix17 localization changes could not be detected. The alignment of human MNRR1, CHCHD10, and yeast Mix17 (Figure 1()) shows a highly conserved region in the hydrophobic central domain of the protein. The identification of several disease-associated mutations in this region and the in silico prediction of a membrane-binding function for this domain [18] suggest that this highly conserved region is necessary for the role of both proteins in a key process for mitochondrial function that can be activated in response to different conditions. An example of a protein-specific change is the Tyr residue present only in MNRR1, which lies just outside of this region. The residue contains a predicted site for tyrosine phosphorylation (http://www.cbs.dtu.dk/services/NetPhos/) and is discussed in the next section. The known functions and properties of MNRR1 and CHCHD10 are compared in Table 1.

## 2. MNRR1 Function

Although MNRR1 was originally picked up in a screening study designed to identify new genes that affect oxidative phosphorylation [19], recent evidence shows that MNRR1 is a biorganellar protein found in both the mitochondria and the nucleus. Interestingly, it appears to have a different function in each compartment: in the mitochondria, it binds to COX and in the nucleus it functions in the transcriptional regulation of genes that contain a highly conserved promoter motif termed the oxygen-responsive element (ORE) [3]. Loss and gain of function experiments have shown that MNRR1 also regulates mitochondrial membrane potential, production of reactive oxygen species (ROS) levels, and cellular redox state [3].

### 2.1. Mitochondrial Function

Regulation of respiration in the mitochondria by MNRR1 has been shown to require its binding to COX [3, 20]. Depletion of MNRR1 results in pleiotropic effects that include an about 50% reduction in cellular oxygen consumption, two-fold-increased ROS levels, 2-fold slower growth [3], and a fragmented mitochondrial phenotype as is associated with stress [21–23]. The binding of MNRR1 to COX is promoted by its phosphorylation at Tyr-99, a reaction that is carried out by ABL2/ARG kinase [20]. ABL2/ARG is a nonreceptor tyrosine kinase that was previously found or predicted to be in the cytosol and nucleus (http://compartments.jensenlab.org/) [24] and now also the mitochondria [20], where MNRR1 is currently the only known mitochondrial target.

### 2.2. Nuclear Function

In the nucleus, MNRR1 is the activator protein in the triad consisting of itself, RBPJκ, and CXXC5, identified on a yeast one-hybrid screen of proteins that specifically interact with the conserved ORE [25]. We have previously shown in reporter assays that MNRR1 activates the promoters for COX subunit 4 isoform 2 (*COX4I2*), as well as itself [3]. Mutation of this element reduces the transactivation potential of the reporter [26]. The current model for the role of MNRR1 in the nucleus is that, under low oxygen tension, it displaces the inhibitory factors from the docking protein RBPJκ to facilitate transactivation.

Depletion of MNRR1 has also been shown to reduce cellular growth rate [3]. MNRR1 knockdown studies in cells have shown a reduction in levels of Atg7, a protein required for the fusion of the vacuolar membrane during autophagy and some subunits of mitochondrial complex I [3], consistent with the effect of MNRR1 reduction in other systems [19].

MNRR1 has been identified as a negative regulator of the mitochondrial apoptotic pathway. A study by Liu et al. [27] revealed that MNRR1 binds to the antiapoptotic protein Bcl-xL under normal physiological conditions and inhibits the accumulation of the proapoptotic protein Bax in the mitochondria. However, under stress conditions, mitochondrial levels of MNRR1 are reduced followed by increased Bax and Bak oligomerization, leading to apoptosis. Currently, there is a paucity in the understanding of how the interaction between MNRR1 and Bcl-xL regulates inhibition of Bax activation. The authors hypothesized that, in addition to being a key player in regulating apoptosis in mitochondria, MNRR1 may have an additional role in the cytoplasm or nucleus.

Cell migration is another function that has been linked to MNRR1 [28, 29]. Overexpression of MNRR1 promotes cell migration in a cell culture-based migration assay, whereas reduced motility is observed upon knockdown of the endogenous protein [29]. Interestingly, analysis of the functional domain revealed that neither the CHCH motif alone nor replacement of a predicted Ser-45 phosphorylation site could exert cell migration-stimulating activity. MNRR1 was shown to interact with HABP1, suppressing migration, whereas MNRR1 was proposed to stimulate cell migration by activating Akt phosphorylation, which in turn leads to RhoA activation, increased Jnk phosphorylation, and ultimately focal adhesion and actin polymerization [29]. Thus, the activities of MNRR1 and HABP1 were proposed to balance cell migration.

MNRR1 has been shown to prime pluripotent stem cells to differentiate towards a neuroectodermal lineage [64]. MNRR1 was identified as a new marker whose expression significantly varies between human-embryonic stem cells (hESC) and human-induced pluripotent stem cells (hiPSC). MNRR1 directly interacts with SMAD4 and segregates it to the mitochondria, resulting in decreased levels of SMAD4 in the nucleus, where it acts as a transcription factor for many of genes of the TGF*β* signaling pathway. This in turn leads to a reduction in TGF*β* and an increased differentiation toward neuroectodermal lineages. SMAD4 has been known to associate with COX subunit II in the mitochondria to regulate apoptotic response. hiPSC have a reduced level of MNRR1 and have a higher expression of nuclear SMAD4 and increased TGF*β* activity, whereas the pluripotent stem cells have a higher MNRR1 expression. These observations suggest that a direct inverse relationship exists between MNRR1 and the activity of the TGF*β* pathway in pluripotent stem cells.

## 3. CHCHD10

In the recent years, high-throughput mass spectrometry analysis has revealed several interacting partners for both MNRR1 and CHCHD10. Unsurprisingly, MNRR1 and CHCHD10 have several common interactors, mostly associated with mitochondrial function such as ETC proteins NDUFS3, NDUFA8 (complex I), COX5A, COX6A1, COX6C (complex IV), and ATP5H (complex V). Other, less intuitive ones include Enoyl-CoA hydratase 1 (ECH1), which is associated with fatty acid metabolism, and complement C1q binding protein (C1QBP), associated with immune function. Both ECH1 and C1QBP are localized to multiple compartments including mitochondria and may play a role in interorganellar communication in conjunction with MNRR1 and CHCHD10. The interaction of MNRR1 and C1QBP has been studied in the context of nonsmall cell lung carcinoma and the network has been predicted to affect cell proliferation, migration, and respiration in cancer cells [28]. An interesting common interactor is USMG5 (upregulated during skeletal muscle growth), also known as DAPIT (diabetes-associated protein in insulin-sensitive tissues). DAPIT is also involved in maintaining ATP synthase (complex V) subunit levels in mitochondria [30]. Although there are no studies linking MNRR1 with diabetes, a CHCHD10 mutation (G66 V) was identified in one family to be associated with adult onset type 2 diabetes [31]. However, as the authors note, additional studies beyond a single family will be needed to confirm an actual disease association.

Both CHCHD10 and MNRR1 have been linked to a number of diseases. For MNRR1, there is a greater number of diseases associated with altered protein levels, whereas in the case of CHCHD10, a number of mutations were associated with disease, particularly neurodegenerative diseases, as well as one case of mitochondrial myopathy (Table 2). This observation, along with the tissue-specific differences in level, may signify that both proteins, though highly similar, are necessary under different conditions. One such condition is the presence of different oxygen levels throughout the body and it is possible that both proteins work together in order to fine-tune mitochondrial function.

## 4. Hypoxic Regulation by MNRR1

Oxygen is critical to cellular physiology. Once absorbed by the lungs, it diffuses into the blood, bound to hemoglobin in the red cells. Delivery of oxygen to the tissues via the circulating blood is finely regulated depending on their metabolic requirements. The partial pressure of oxygen (pO_2_) is widely used to indicate the amount of oxygen in a particular tissue. In a clinical setting, the units for the pO_2_ are mm Hg and in an experimental setting, the units are percent O_2_. Under physiological conditions, the pO_2_ in human tissues ranges widely between and within mammalian tissues (reviewed in [32]) but is well below those used in standard cell culture experiments. For example, relatively low in vivo oxygen levels were found in the bone marrow of mice, ranging from 11.7 to 31.7 mm Hg (1.5–4.2% O_2_) with an average value of 20.4 mm Hg (2.7% O_2_) [33]. Intermediate levels of 5% O_2_ (37.8 mm Hg) were reported to be optimal for myogenic commitment of muscle stem cells [34], while higher average levels ranging from 29.7 to 61.8 mm Hg (3.9–8.2% O_2_) were reported in the mouse brain [35]. In sharp contrast, for the oxygen tension in a standard experimental cell culture setting, the pO_2_ is ~20%. Generally, in the experimental cell culture setting, a reduction in the levels of oxygen from the ~20% standard is termed hypoxia. Obviously, ~20% oxygen is hyperoxic in comparison to in vivo oxygen tensions, and great caution should be taken when extrapolating conclusions derived from cell culture work to the in vivo situation.

With a reduction in the available oxygen, a cell activates multiple signaling pathways in an attempt to maintain homeostasis. The most widely studied hypoxic regulators are the HIFs (hypoxia-inducible factors). Two distinct factors, HIF1*α* and HIF2*α*, have received the most attention. In cell culture models, HIFs are stabilized at an oxygen tension of 1-2% or lower [26, 36–38]. However, a study by Holmquist-Mengelbier et al. has shown HIF2*α* stabilization at a moderate level of hypoxia (5% O_2_) in a neuroblastoma cell line [39], an effect observed only when the cells are maintained under chronic hypoxia (72 h). HIFs, once stabilized, translocate to the nucleus to bind the hypoxia-responsive elements (RCGTG) in the promoters of genes they will activate. An additional family member, HIF3*α*, has come to light in recent years. HIF3*α* has multiple spliced variants, with HIF3*α*2 being induced under hypoxia. The most intriguing part is that HIF3*α* functions as an inhibitory factor for HIF signaling [40].

In addition to being a transcription factor under hypoxic conditions, HIF1*α* was shown in a breast cancer model to be instrumental in activating *γ*-secretase by interacting with and repositioning the catalytic subunit [41]. Several groups have shown HIF-independent pathways to play a key role in the regulation of hypoxia-responsive genes. HMG1.2 has been shown to be one such gene in *Caenorhabditis elegans* that binds promoter DNA at low oxygen tensions. In addition, mammalian transcriptional regulators such as c-Myc [42], ATF-4 [43], and NF-*κ*B [44] have also been shown to function in hypoxia in a HIF-independent manner. Other signaling pathways, such as mTOR [45], are also regulated under hypoxia in a HIF-independent manner. Thus, how does a cell respond to oxygen tensions that are low but not sufficiently so to stabilize HIFs?

**Figure 2:**
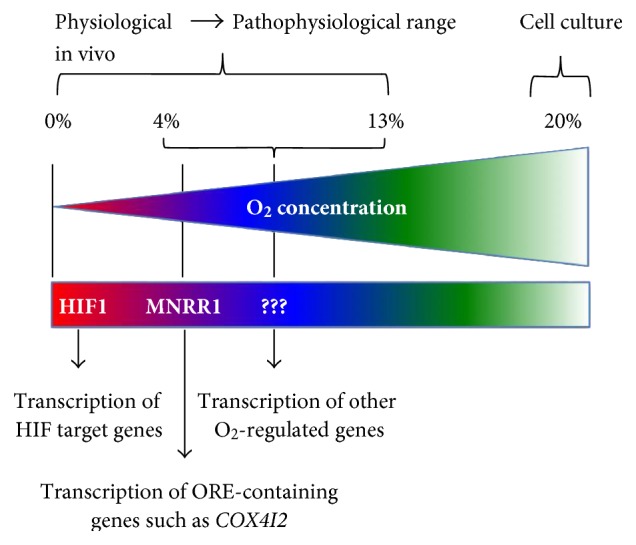
Model for transcriptional response to decreasing oxygen levels. The model proposes that, as tissue oxygen levels decrease from the artificial 20% level typically used for tissue culture, different transcriptional programs come into play to try to achieve homeostasis.

We have previously shown MNRR1 to be a biorganellar regulator. In addition to its localization and function in the mitochondria, this protein is also localized to the nucleus, where it binds a conserved 13 bp DNA sequence, the ORE, along with RBPJ*κ*. MNRR1 activates the ORE by displacing the inhibitory factor CXXC5 that is bound to RBPJ*κ*. This ORE is also independent of the HIFs and is maximally active in a cell culture system at 4% oxygen, in marked contrast to a reporter with HIF-binding elements that is maximally active at oxygen tensions of ≤1% [26]. Although 4% oxygen in an experimental system is hypoxic, considering the oxygen tension in the human body, it could be that MNRR1 is the basal transcriptional factor for genes harboring the ORE in organs that have an oxygen tension of ~4% (Figure 2()).

Every cell in the body is exposed to an oxygen tension that may vary from cell to cell or organ to organ. A regulatory system is required to cope with differential oxygen tensions, to induce a transcriptional program that would lead the cell towards normalcy, that is, to achieve its normal homeostatic state. It is logical to hypothesize that cells induce a distinct regulatory signature at a very specific oxygen tension. We propose, based on the available literature, that MNRR1 under moderate hypoxia, and HIFs under severe hypoxia, carry out these functions. Identification of the factors that play a key role at distinct oxygen tensions, specifically other CHCH domain-containing proteins, and their mechanism of regulation will undoubtedly be of considerable importance in understanding signaling pathways involving the mitochondria of a cell under hypoxic stress.

## 5. MNRR1 and Disease

MNRR1 has been associated with a number of diseases, most commonly Parkinson's (PD) and Lewy body diseases [46–48]. The association with PD is difficult to pin down, however. First, the associations found are quite rare: although a number of studies have identified nonsynonymous amino acid changes that have a higher frequency in patients with PD than in controls, the absolute population frequency is <<1%. Furthermore, some studies have not found the reported amino acid changes or have not found them at a higher level in patient than in control populations [49–55]. Second, few studies have involved extended families that allow tracking the segregation of putative mutations among affected and unaffected members. Lastly, the disease associations have been based on allele frequencies and lack a mechanistic basis for their pathological action.

Funayama et al. carried out a large study with Japanese populations [47]. They identified a missense mutation in MNRR1 (T61I) in a family by next-generation sequencing, then obtained samples from an additional 340 patients with autosomal dominant (AD) PD, 517 patients with sporadic PD, and 559 controls. Three MNRR1 mutations in four of 341 index cases from independent families with ADPD were detected: T61I, R145Q, and a splice site mutation. Of these, the T61I mutation is notable because it was not present in control populations and because it cosegregated in a Japanese family with ADPD. These studies are important in that they show segregation with Parkinson's disease in a family as assessed by Sanger sequencing.

Lewy body diseases (LBD), a form of dementia that includes PD, were also targeted for a study of MNRR1 sequence variants [48]. More than 1600 patients from the US, Ireland, and Poland had PD, 610 had a non-PD LBD, and altogether 1432 were controls. The T61I variant, however, was not found in this study, and other coding region variants were found a maximum of 3 times among the pooled 2237 patients compared to 0 or 1 time among the various control groups.

The rarity of MNRR1 variants among PD patients in all the studies taken together, along with the presence of most variants also in nonsymptomatic controls, raises the question of whether MNRR1 is indeed a risk factor for PD. It will require mechanistic—including animal—studies to address this question.

MNRR1 has been examined for association with other genetic diseases. One study sought associations with multiple system atrophy (MSA) and amyotrophic lateral sclerosis (ALS) in Han Chinese patients, based on previous detection of common genetic factors [56]. All four exons of MNRR1 were sequenced after PCR amplification in 89 MSA patients, 424 sporadic ALS patients, and 594 controls. No exonic variant was detected in the MSA patients; four were detected in 6 ALS patients, including P2L and S85R present in PD patients; however, P2L was present at about an equal frequency in controls without neurological disease and S85R was present in 1 patient and 0 controls. Thus, genetic variants of MNRR1 did not appear important in MSA or ALS in this population.

The neurological connection to MNRR1 was further explored in several other conditions, one of which was Huntington's disease (HD). Human pluripotent stem cell (hPSC) lines were generated containing the mutant *huntingtin* (*HTT*) gene to explore early developmental changes in gene expression [57]. Both human-embryonic stem cells (hESCs) and differentiated neural stem cells (NSCs) were examined. One of the three genes whose expression differed significantly from wild-type cells in both hESCs and NSCs was MNRR1, for which also corresponding protein level differences were confirmed. Dysregulation of MNRR1 was previously observed in blood cells from HD patients [58]. In both cell types, MNRR1 increased with differentiation but more so in *HTT* mutant cells. Since HTT interacts with both mitochondrial metabolism via an effect on PGC-1*α* activation and production [59] and cell migration [29], a role in neuronal differentiation is not surprising although the precise nature of that role has yet to be clarified.

MNRR1 was further connected to neurological development when it was shown to be downregulated in iPS cells derived from patients with lissencephaly, a congenital brain malformation caused by defects in neuronal migration [60]. The iPS cells were generated from two patients; one contained a chromosome 17 microdeletion that includes LIS1, a known microcephaly gene [61]. The other contained a missense mutation in *TUBA1A*, another gene associated with cortical migration disorders [62]. Since both genes are associated with iPS cells generated from both patients and MNRR1 has been shown to be relevant to cell migration [29], Shimojima et al. examined the expression of MNRR1 in patient and control iPS cells undergoing neural differentiation [60]. Control cells increased MNRR1 expression at 8 and 16 days whereas cells from both patients started with a lower level of expression and only marginally increased it with time. The association noted above between MNRR1 and huntingtin, of MNRR1 and cell migration [29], and of huntingtin with microtubules [63], suggests that MNRR1 could be involved in neuronal migration. Furthermore, MNRR1 has also been suggested to prime the differentiation potential of human iPS cells to neuroectodermal lineages [64] and to inhibit apoptosis [27], an important component of normal brain development [65]. Taken together, there is ample reason to connect MNRR1 with cortical development but clearly this area is in need of further investigation.

Finally, MNRR1 has been connected to tumorigenesis. One report shows that it is coamplified with the epidermal growth factor receptor (EGFR) in nonsmall cell lung carcinoma (NSCLC) [28]. Protein levels of MNRR1 and EGFR protein are upregulated in NSCLC tumor-derived xenografts as compared to those of the normal lung. Experiments on proteome changes in NSCLC cells upon MNRR1 knockdown suggest that MNRR1 gene copy number and protein levels are linked with EGFR as a driver in NSCLC. Moreover, the MNRR1 knockdown in NSCLC cells alleviates cell proliferation, migration, and mitochondrial respiration. Examination of protein-protein interactions of MNRR1 revealed two interactome hub proteins, C1QBP/HABP1, a mitochondrial protein, and YBX1, an oncogenic transcription factor. The nature of these linkages will need to be better defined.

MNRR1 has also been connected to liver carcinogenesis (HCC) via the effect of hepatitis C virus nonstructural protein 2 (NS2) on upregulating the expression of MNRR1 [66]. MNRR1 was highly stained in a biopsy of liver cancer but not in the adjacent normal tissue. Furthermore, in examining histological biomarkers for HCC, MNRR1 was found highly expressed in >95% of samples. Whether altering its expression level can alter markers of tumorigenesis awaits further studies. Furthermore, it was revealed that c-AMP response element binding protein (CREBP) plays an important role in the transcriptional activation of MNRR1. Owing to the complexity of MNRR1 function, it is likely to be controlled at many levels. The mechanisms that control the expression of MNRR1 are yet to be clearly understood.

## 6. CHCHD10 and Disease

The CHCHD10 isoform is a 142 amino acid protein. CHCHD10 was originally picked up in a screen using the guilt by association approach to be highly enriched in the heart and skeletal muscle [67]. This study also confirmed that CHCHD10 is a mitochondrial protein, and a transient knockdown in HeLa cells decreased both COX activity and ATP levels to ~50% of wild-type cells. Within the mitochondria, CHCHD10 localizes to the intermembrane space [68] and interacts with the members of the mitochondrial contact site and cristae organizing system (MICOS) complex, whose stability may also require CHCHD3 and CHCHD6 [69].

CHCHD10 has been linked to a number of neurodegenerative disorders in the past few years. The first study identified and characterized a mutation, S59L, to be associated with a frontotemporal dementia- (FTD-) amyotrophic lateral sclerosis (ALS) phenotype [68]. Since then, several mutations in CHCHD10 have been associated with neurodegenerative disorders such as ALS and one was linked to a mitochondrial myopathy. Table 2 summarizes the mutations identified so far; their clinical implications have been well summarized in a recent review [70]. Although over 10 different mutations have been discovered, few of them have been analyzed in detail and causally associated with the phenotypes seen. Three have been tested thus far in cell culture model systems to elucidate the effects of these mutations, S59L, P34S [68, 69], and R15S/G58R [71]. Bannwarth et al. identified the S59L mutation in a family of French origin. They also determined the effects of this mutation in skin fibroblasts obtained from two patients and found that the mitochondria were decreased in length, had altered cristae morphology, and showed defects in MICOS assembly and nucleoid formation [68, 69]. The same group also functionally characterized the S59L mutation along with another, P34S. Overexpression of both these CHCHD10 mutants led to altered cristae morphology. The only other CHCHD10 mutation assessed in a cell culture system is the double-mutation R15S in cis with G58R, identified in a family of Puerto Rican origin [71]. The authors investigated its effects in cells overexpressing this mutant protein and found that it led to a loss of mitochondrial networks, forming smaller, more punctate mitochondria. In comparing this double mutant with individual R15S or G58R mutants, they found that the G58R mutant is sufficient to cause the altered phenotype. Hence, they concluded that the R15S mutation may not be pathogenic and the effects seen may be only due to the G58R mutation.

Since the original discovery of mutations in CHCHD10 linked to ALS, many screens were conducted across different populations to detect other harmful mutations in CHCHD10. Despite the seemingly large number of mutations identified, all the variants may not necessarily contribute to disease. There have been cases where the same mutation is found in both the disease patients and in control groups, leading to speculation whether the mutation is pathogenic, a risk factor, or a benign polymorphism. For example, the P34S mutation is associated with ALS and FTD by several studies [72–74], but the same mutation has been identified in healthy control individuals and hence is considered nonpathogenic [75–78]. One shortcoming may be that some of the ALS studies did not consider a sufficient number of control individuals [9, 78]. However, another problem with the interpretation of the data is the age of disease onset, which for neurodegenerative disorders is often relatively late. Therefore, individuals classed as controls may actually be ones where the disease has not manifested yet, leading to a premature classification of the mutation as nonpathogenic. As a result, it seems essential to ensure age-matched control and affected populations as well as to estimate from follow-up studies the proportion of controls who change status after initial data collection. Lastly, there is at least one case where the identified mutation is incorrect due to improper annotation of the gene [9]. The canonical CHCHD10 protein sequence (UniProtKB) has 4 cysteines at positions 102, 112, 122, and 132 which are connected by 2 disulfide linkages to form the CHCH domain. Any mutation in one of these critical cysteines is likely to lead to protein misfolding. One study has identified a mutation of Glu-102 to His whereas, in the canonical protein, position 102 is a cysteine and part of the twin CX_9_C motif [16]. The same group also found a mutation at Tyr-92 whereas the original residue in the CHCHD10 sequence is alanine. The template sequence that was analyzed for identifying mutations is a 149-amino acid sequence expressed from a splice variant (ENST00000401675.7) for which the protein status is unreviewed on UniProtKB (B5MBW9).

Despite the presence of such confounding data, some mutations such as G66V have been identified exclusively in patient populations. The G66V mutation has been associated with a diverse spectrum of disorders including ALS [79, 80] and motor neuron disease [81, 82]. In one study, it was seen that, even within a single family where all the affected individuals carried the G66 V variant, many different phenotypes were displayed ranging from CMT2-type axonal neuropathy to spinal muscular atrophy that presented as an ALS-like disease [31]. Another mutation, P80L, is almost exclusively present in patients with ALS [75, 76, 83]. The P80L mutation recently was seen in one control subject, but the authors state that this subject was 57 years of age at the time of the study and may develop symptoms at a later age. They concluded that the P80L mutation might be a pathogenic one with reduced penetrance [84]. What is suggested by this data is that (1) the association with disease is not always clear and (2) different mutations may have different penetrance in different individuals. Some of these issues will be clarified by the study of CHCHD10 function in the cell both under physiological and pathological conditions.

## 7. Other Genes That Harbor the ORE in Their Promoters

The oxygen-responsive element (ORE) is a 13 bp sequence originally identified in the promoter of *COX4I2*, one of the subunit isoforms of COX. The transcription of genes from the ORE is regulated by 3 proteins, RBPJκ, CXXC5, and MNRR1 [25]. Genes containing the ORE are a target for transcriptional activation by MNRR1, which includes MNRR1 itself [3]. A systematic in silico analysis of human genes containing the ORE identified 28 genes containing the ORE derived from *COX4I2* or MNRR1 upstream of the first exon. These are listed in Table 3. Many of the genes in the list are yet to be characterized (*LOC105370119*, *RBBP8NL*, *KIAA1614*, *ADPRHL1*, *NOL9*, *C18ORF8*, *C2CD2*, and *RNF150*), or are microRNA genes (*MIR36481*, and *MIR661*), long noncoding RNA genes (*LINC00403*), or pseudogenes (*EEF1DP3*), and hence cannot be classified into any major category for cell function.

The genes on the list whose function has been characterized to some extent have interesting implications. The target list includes genes that control mitochondrial function such as *SDHAF1* (succinate dehydrogenase assembly factor 1), a complex II assembly factor, and *FBP1* (fructose bisphosphatase 1), an enzyme that regulates gluconeogenesis (Lamont 2006). Other target genes encode proteins such as *MADCAM1* (mucosal vascular addressin cell adhesion molecule 1), *MARCKSL1* (macrophage myristoylated alanine-rich C kinase substrate-like 1), and *CDH4* (cadherin 4), which are associated with cell adhesion and migration, a process known to be regulated by MNRR1 [29] and *LACTB *(lactamase beta), which forms filaments in the mitochondrial IMS and is part of a network of genes that were validated to have a casual association with obesity traits [85]. Another putative MNRR1 target gene is *USP28* (ubiquitin-specific peptidase 28), which encodes a deubiquitinating enzyme that contributes to DNA damage-induced activation of apoptosis [86], another key pathway with which MNRR1 is associated [27].

Other ORE-harboring genes include some that may affect neuronal and CNS function but require further characterization. *WWC1* plays a role in Hippo/SWH signaling [87] and variants of this protein have been associated with memory performance and lipid binding [88]. *CNPY4* is a transcriptional inhibitor that modulates FGF signaling in the midbrain-hindbrain region in the zebrafish model system [89]. ADRA2A is a protein belonging to the GPCR family and is involved in the regulation of neurotransmitter release from adrenergic neurons in the CNS [90].

## 8. Conclusion

**Figure 3:**
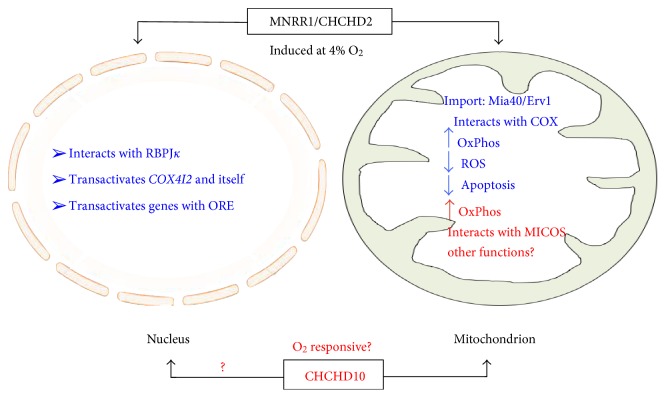
Model for MNRR1 function. The model shows the known functions of MNRR1 in both the nucleus and the mitochondria. Whether CHCHD10 functions similarly remains to be determined.

CHCHD10 and MNRR1 are both important proteins that regulate cell growth and metabolism. The functional studies regarding MNRR1's role as biorganellar regulator of oxidative phosphorylation [3], and the characterization of a posttranslational modification [20], provide clues to identify the role of this protein in cellular function and pathways that can be targeted in order to regulate its levels under hypoxic stress conditions. Similar studies are necessary for CHCHD10. Since hypoxia is associated with so large a proportion of diseases, it is not surprising that disease-associated variants are coming to light. One important corollary that can be drawn from the high similarity between the two proteins and the fact that both have a common ancestor is that both proteins would be part of a similar process [14]. It would be tempting to speculate that during the course of evolution, when the ancestral gene was duplicated, both copies underwent distinct changes, giving rise to two separate genes, perhaps in order to respond to different conditions but to regulate one critical process—oxidative phosphorylation—that is vital for cell survival. Hence, basic mechanistic studies in the case of CHCHD10, and further studies for MNRR1, would provide a platform for identifying the effects of both these proteins individually and as part of a system of regulation in response to different stress conditions including but not limited to hypoxia (Figure 3()).

An interesting possibility is that each of the CHCH domain-containing proteins is responsive at distinct experimental oxygen tensions. If true, this would provide a mechanism, together with the HIF system, to adapt and fine-tune cellular responses to the wide range of oxygen concentrations found under physiological and pathological conditions. Furthermore, one can ask whether oxygen tension is the sole regulator for CHCH domain-containing proteins or whether there are other conditions that affect their function, as for example shown for MNRR1 tyrosine phosphorylation. Finally, it will be critical to further identify and characterize the mutations associated with these proteins so that they could be exploited clinically in diagnosis as well as treatment.

## Figures and Tables

**Table 1:** Comparison of various identified functions, effects, and properties of MNRR1 and CHCHD10.

	MNRR1	CHCHD10
Protein length	151	142
CHCH domain	114–144	102–132
Interactions identified using mass spectrometry (BioGRID database)	97 total unique interactors (common interactors for both: C1QBP, NDUFS3, NDUFA8, COX5A, COX6A1, COX6C, ATP5H, ECH1, USMG5)	42 total unique interactors (common interactors for both: C1QBP, NDUFS3, NDUFA8, COX5A, COX6A1, COX6C, ATP5H, ECH1, USMG5)
Expression (Human Protein Atlas)	Expressed in all tissues at medium to high levels	Muscle, heart, liver (high), brain (medium), and low levels for other tissues
Mitochondrial function	Regulation of COX activity, ROS production [3], apoptosis [27]	Regulation of COX activity and ATP production [67], cristae morphology [68, 69]
Nuclear function	Transcriptional activator for *COX4I2* and itself [3]	Not known to be localized to nucleus
Hypoxia sensitivity	Upregulated at 4% oxygen [25]	Unknown
Posttranslational regulation	Phosphorylated at Y99 by Abl2 kinase which activates mitochondrial function [20]	Unknown
Disease association (altered protein/transcript levels)	Huntington's disease [57], hepatocellular carcinoma [66], nonsmall cell lung carcinoma [28], lissencephaly [60]	Gastric cancer [91]
Mutation in protein associated with disease	Parkinson's disease [47]	Mitochondrial myopathy, amyotrophic lateral sclerosis, Alzheimer's disease, frontotemporal dementia, cerebellar ataxia, spinal muscular atrophy, Charcot-Marie-Tooth disease type 2A, motor neuron disease (specific references and mutations summarized in Table 2)
Functionally characterized mutations	Q112H [20], 300+5G>A [47]	S59L and P34S [68, 69], R15L/G58R [71]

**Table 2:** Mutations identified in CHCHD10 associated with neurodegenerative disorders and mitochondrial myopathy.

Mutation	Disease	Reference
Pro12→Ser^∗^	ALS	[76]
Arg15→Leu	ALS, motor neuron disease	[72, 75, 79, 81]
His22→Tyr	Behavioural variant FTD	[92]
Pro23→Thr/Ser/Leu	FTLD (T), behavioural variant FTD (S), semantic dementia (L)	[75] (T); [76] (S); [92] (L)
Pro34→Ser	FTD-ALS, ALS	[73, 74, 76]
Ala35→Asp	FTLD, Alzheimer's disease	[75, 93]
G58→Arg (in cis with Arg15→Ser)	Mitochondrial myopathy	[71]
Ser59→Leu	FTD-ALS, cerebellar ataxia	[68, 73]
Gly66→Val	ALS, LOSMoN/SMAJ, motor neuron disease, CMT2A	[79–82]
Pro80→Leu	ALS	[75, 83, 76]
Gln82→X	Atypical FTD with Parkinsonism	[76]
Tyr92→Cys^∗∗^	ALS	[9]
Pro96→Thr^∗^	ALS	[76, 94]
Gln102→His^∗∗^	ALS	[9]
Gln108→X^∗^	Atypical FTD and Parkinson's disease	[84]

^∗^Found outside exon 2. ^∗∗^Incorrectly assigned mutations in canonical CHCHD10.

**Table 3:** List of genes containing the oxygen-responsive element (ORE) identified using Geneious (www.geneious.com). ORE sequences for MNRR1/CHCHD2 and COX4I2 in the table were used as reference sequences and searched against the human genome (GRCH38/hg38). Matches of 83.5% or above within 1000 bp 5′ to the start of translation were listed.

ORE	Genes containing ORE up to 1000 bp upstream of the gene
*MNRR1* (5′-TGTCCCACGTCCGGA-3′)	*LOC105370119*, *MIR661*, *MNRR1*, *ST18*, *MADCAM1*, *RBBP8NL*
*COX4I2* (5′-TTCCCACGCTGGGG-3′)	*ADPRHL1*, *ADRA2A*, *C18orf8*, *C2CD2*, *CASZ1*, *CDH4*, *CNPY4*, *COX4I2*, *EEF1DP3*, *ESYT1*, *FBP1*, *KIAA1614*, *LACTB*, *LINC00403*, *MAP2K5*, *MARCKSL1*, *MIR36481*, *NOL9*, *RNF150*, *SDHAF1*, *USP28*, *WWC1*
